# Biopsychosocial and Cultural Determinants of Functioning and Healthcare Outcomes in Chronic Non-Cancer Pain: An Integrative Review

**DOI:** 10.3390/healthcare14060725

**Published:** 2026-03-12

**Authors:** Rocío Cáceres-Matos, Miguel Garrido-Bueno, Juan Manuel Fernández-Sarmiento, Ana María Porcel-Gálvez, Manuel Pabón-Carrasco

**Affiliations:** 1 Research Group PAIDI-CTS-1050: “Complex Care, Chronicity and Health Outcomes”, Faculty of Nursing, Physiotherapy and Podiatry, University of Seville, 41007 Sevilla, Spain; rcaceres3@us.es (R.C.-M.);; 2 Red Cross Nursing University Center, University of Seville, 41004 Sevilla, Spain

**Keywords:** chronic pain, models, biopsychosocial, mental health, social factors, quality of life, integrative review

## Abstract

**Background:** Chronic non-cancer pain (CNCP) is an increasing global health concern and a multidimensional condition shaped by biological, psychological, social, and cultural factors, with impacts on functioning, quality of life, and healthcare. However, evidence remains fragmented, limiting integrated understanding and care. Objective: This study aimed to synthesize and critically analyze existing evidence on the biological, psychological, social, and cultural dimensions characterizing individuals with CNCP, and their impact on functionality, quality of life, and healthcare. **Methodology:** An integrative review was conducted following the Whittemore and Knafl framework. Searches were performed in Medline, Cumulative Index of Nursing and Allied Literature Complete (CINAHL), PsycINFO, Scopus, Web of Science, and grey literature in English and Spanish, without time restrictions. Studies were screened using predefined eligibility criteria and appraised with Joanna Briggs Institute tools. Data were systematically extracted and synthesized using thematic analysis to identify key attributes of people living with CNCP. Quantitative findings were summarized descriptively and mapped to thematic domains, while qualitative data were analyzed interpretively. Both evidence streams were integrated through convergent thematic synthesis. **Results:** Forty-four studies were included, predominantly cross-sectional and observational. Five themes emerged: biological aspects; functioning and quality of life; psychological and mental factors; social support and peer relationships; and social and gender determinants. CNCP was consistently associated with multimorbidity, sleep disturbance, psychological distress, and maladaptive coping, contributing to reduced functional capacity, greater disability, poorer quality of life, and increased healthcare utilization. Socioeconomic disadvantages and environmental constraints were linked to higher pain burden, whereas resilience and social support emerged as protective factors mitigating functional and psychosocial impact. **Conclusions:** Evidence largely concentrates on biomedical, functional, and psychological dimensions, whereas social determinants and healthcare quality remain comparatively underexplored. Broadening these perspectives is essential to inform public health strategies and support multidisciplinary, equitable care for individuals living with CNCP.

## 1. Introduction

Chronic Non-Cancer Pain (CNCP) has emerged as a major global public health challenge, defined as persistent or recurrent pain lasting more than three months and not attributable to malignant processes. Unlike acute pain, which serves a protective physiological function, CNCP is now formally recognized as a diagnostic entity following the implementation of the ICD-11 in 2022, reflecting a paradigm shift toward conceptualizing chronic pain as a condition with distinct biological, psychological, and social dimensions [1].

The prevalence of CNCP is high, with estimates varying according to diagnostic criteria and population characteristics [2,3]. Chronic pain remains one of the leading contributors to years lived with disability globally [4]. In Europe, prevalence ranges from 12% to 48%, with higher rates consistently observed among women and older adults [2]. In Spain, recent epidemiological evidence indicates that 25.9% of adults experience CNCP [5]. These variations reflect the influence of demographic, methodological, and health system factors on prevalence estimates, as well as broader socioeconomic inequalities across populations [6].

CNCP exerts a multidimensional impact. Biologically, recent research highlights mechanisms such as central sensitization, altered neuroplasticity, and dysregulated inflammatory pathways as contributors to pain chronification [7]. Physically, CNCP restricts mobility, reduces functional capacity, and generates progressive disability [4,7]. Psychologically, it is strongly associated with anxiety, depression, emotional dysregulation, and cognitive vulnerability, with these factors acting both as consequences and drivers of persistent pain [8,9]. Many individuals report frustration, helplessness, and social isolation, often exacerbated by stigma and limited public understanding [10].

From a socioeconomic perspective, CNCP generates substantial healthcare expenditure, including repeated consultations, diagnostic procedures, pharmacological treatment, and complementary therapies [10,11]. Its impact extends to families and caregivers, contributing to financial strain and diminished quality of life [7]. Moreover, social determinants, including socioeconomic status, social support, and working conditions, play a decisive role in pain experience and access to care, consistent with emerging multilevel and life-course perspectives on pain inequities [12,13].

Current evidence shows that CNCP emerges from the interaction of biological vulnerability, psychological adaptation, social context, and cultural meaning. These dimensions shape functional outcomes, coping strategies, quality of life, and healthcare utilization, underscoring the need to move beyond biomedical models towards a truly integrative approach [9,12].

Despite growing recognition of its complexity, CNCP care remains fragmented, with limited access to multidisciplinary pain management programs and inconsistent integration of psychological and rehabilitative interventions. Pharmacological treatment, particularly opioid prescribing, remains prevalent despite modest long-term effectiveness and well-documented risks [13].

Given this multidimensional nature of CNCP, understanding how biological, psychological, social, and cultural factors shape patients’ functioning, quality of life, and healthcare use is essential. Therefore, the aim of this study was to synthesize and critically analyze existing evidence on the biological, psychological, social, and cultural dimensions characterizing individuals with CNCP, and their impact on functionality, quality of life, and healthcare. In particular, the review focuses on identifying key biopsychosocial determinants and consequences associated with CNCP and how these influence functional outcomes and care experiences. Furthermore, the synthesis seeks to integrate these dimensions within a biopsychosocial perspective to contribute to a more coherent conceptual understanding of chronic non-cancer pain.

## 2. Materials and Methods

An integrative review was conducted to synthesize empirical and theoretical evidence on the experience of individuals with CNCP. The review adhered to the recommendations of the Sex and Gender Equity in Research (SAGER) guidelines to ensure the appropriate consideration of gender aspects in the reporting process [14].

This methodological approach was selected because integrative reviews allow the inclusion and synthesis of evidence from diverse study designs, including quantitative, qualitative, and theoretical literature. Given the multidimensional nature of chronic non-cancer pain, an integrative review was considered more appropriate than a conventional systematic review, which typically focuses on more narrowly defined research questions and homogeneous study designs [15]. The framework proposed by Whittemore and Knafl was therefore adopted to support a comprehensive and structured synthesis of heterogeneous evidence.

The protocol of this review was registered prospectively in Open Science Framework (registration doi: https://doi.org/10.17605/OSF.IO/9G5PN) [16].

The structured methodology for integrative reviews proposed by Whittemore and Knafl [17], which comprises five sequential stages, was followed: (1) problem identification; (2) literature search; (3) data evaluation; (4) data analysis; and (5) presentation [15]. Integrative reviews are designed to synthesize both empirical and theoretical literature, thereby providing a comprehensive understanding of a phenomenon or healthcare problem [18].

### 2.1. Problem Identification

According to the first stage of the Whittemore and Knafl framework, the problem identification phase requires defining the phenomenon of interest and clarifying its conceptual boundaries [17]. The review was guided by the research question: “In individuals living with chronic non-cancer pain, what dimensions and factors related to their health and care experience are described in the scientific literature?” The research question was structured using the Person, Exposure, Outcome (PEO) framework, a widely recognized variant of the PICO model [19], which facilitated the identification of defining attributes.

### 2.2. Search Strategy and Identification

In the second stage of the Whittemore and Knafl framework, a comprehensive literature search was performed to identify empirical and theoretical sources relevant to the phenomenon under study [17]. This process aimed to ensure breadth and depth in data collection and to minimize selection bias.

A systematic search strategy was developed and agreed upon by the research team as follows: (“Chronic Pain” OR “persistent pain” OR “long term pain” OR “Widespread Chronic Pain”) AND (“Excessive anxiety” OR “Excessive fear” OR “Impaired intestinal elimination” OR “Impaired mood regulation” OR “Impaired physical mobility” OR “Ineffective sleep pattern” OR “altered ability to continue activities” OR “Altered sleep-wake cycle” OR “Evidence of pain using standardized pain behavior checklist for those unable to communicate verbally” OR Fatigue OR “Hypervigilance to pain” OR “Verbal report of pain”) AND Adult NOT (Oncology OR Metastasis OR “Cancer Pain”). Truncation and Boolean operators were used to refine results from original searches.

The search strategy incorporated both conventional biopsychosocial constructs and selected standardized diagnostic labels derived from nursing taxonomies (e.g., NANDA-I) to operationalize complex experiential domains of chronic non-cancer pain [20]. These terms were not used as diagnostic outcomes, but as sensitizing concepts to capture functional, emotional, and behavioral manifestations frequently underrepresented by broader constructs.

Searches were conducted in both peer-reviewed and grey literature, covering studies published in English and Spanish. The scientific literature was retrieved from Medline, CINAHL, PsycINFO, Web of Science, and Scopus, while grey literature was accessed through OpenGrey, Google Scholar, ProQuest, and websites of national and local health authorities. The process for both peer-reviewed and grey literature was performed between May and July 2025 by two researchers independently, consulting a third one in case of disagreement. Screening in Google Scholar was limited to the first 200 results sorted by relevance, a pragmatic approach commonly used in evidence syntheses given that the most relevant records are typically retrieved within the first results pages [21].

To avoid duplication, the Cochrane Library and PROSPERO were also screened for ongoing or completed reviews on similar topics. An updated search was performed immediately before the analysis, in July 2025, to capture the most recent evidence.

### 2.3. Eligibility Criteria

References were included if they met the following criteria: (1) empirical studies addressing chronic non-cancer pain (CNCP) in adults (≥18 years), providing data on prevalence, associations, or outcomes; (2) literature contributing to the clarification of domains, constructs, or interpretative frameworks related to CNCP; (3) no restrictions on publication date; and (4) publication in English or Spanish.

On the contrary, exclusion criteria considered documents: (1) that addressed chronic degenerative infectious diseases or populations with cognitive impairment; (2) that lacked full-text availability; and (3) of secondary resources without empirical data, such as reviews of literature or editorials. Populations with cognitive impairment were excluded because such conditions may substantially affect the perception, communication, and reporting of pain, as well as the assessment of psychological and social dimensions associated with chronic pain [22]. Although the integrative review methodology proposed by Whittemore and Knafl allows the inclusion of review articles, this study excluded them to prioritize primary empirical research in order to avoid duplication of evidence and potential overlap of findings already synthesized in previous reviews.

### 2.4. Data Extraction and Analysis

All retrieved records were managed using Rayyan [23]. Two reviewers independently removed duplicates, screened titles, abstracts, and full texts, in accordance with the Preferred Reporting Items for Systematic reviews and Meta-Analyses (PRISMA) 2020 guidelines [24]. Discrepancies were resolved through consensus or consultation with a third reviewer expert in CNCP management.

A standardized data extraction form, adapted from the Cochrane Handbook and piloted on five studies, was used to ensure consistency [25]. The extraction process was structured using the PEO framework, whereby participant characteristics (Person), biopsychosocial and cultural attributes related to CNCP (Exposure), and reported consequences (Outcome) were systematically captured. Cultural aspects were interpreted as the socially shared meanings and norms through which chronic pain is understood and experienced. All extracted data were entered into a summary matrix that included author, year, region, design, aim, definition or use of CNCP, identified attributes, related factors or consequences, and measurement tools.

The methodological rigor and strength of evidence of the included studies were assessed using the Scottish Intercollegiate Guidelines Network (SIGN) levels of evidence framework. This hierarchical system classifies studies according to their methodological quality and risk of bias, ranging from high-quality systematic reviews and randomized controlled trials (Level 1) to non-analytic studies and expert opinion (Levels 3–4). Based on these criteria, the studies included in this review were categorized into levels such as 2++, 2+, 3, or 4. In addition, grades of recommendation (A–D) were assigned according to the overall strength and consistency of the evidence [26].

Following the third and fourth stages of the Whittemore and Knafl framework (data evaluation and data analysis), the information extracted from included studies was analyzed using a thematic synthesis approach. The process combined inductive and deductive reasoning, allowing the identification of emerging concepts while preserving alignment with the review objectives [17]. The reviewers independently coded and compared findings from each study using a mixed inductive-deductive strategy, which enabled the identification of both predefined and emerging dimensions of the CNCP experience. Empirical and theoretical sources were appraised and coded separately and subsequently integrated through thematic synthesis to generate higher-order dimensions, in accordance with the Whittemore and Knafl framework [17].

## 3. Results

**Figure 1:**
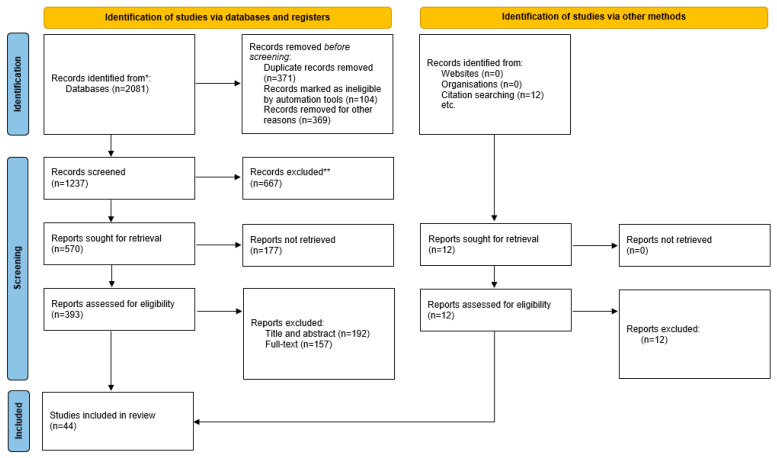
PRISMA 2020 flow diagram that represents the process of selection of the included studies. * The number of records identified from each database or register. ** Indicate how many records were excluded.

Forty-four studies met the inclusion criteria. The search for grey literature did not yield any relevant results to be included in the review. The full selection process was documented using a PRISMA-based flow diagram (Figure 1).

### 3.1. Characteristics of the Included Studies

Most of the studies were published in 2021 (n = 12, 27.27%), followed by 2024 (n = 10, 22.73%), 2022 (n = 9, 20.45%), and 2020 (n = 8, 18.18%). This recent concentration of publications (2020–2025) may reflect growing global interest in CNCP as a multidimensional health concern. The most prevalent levels of evidence were 2+ (n = 34, 77.27%) and 2++ (n = 4, 9.09%). The grades of recommendation were C (n = 34, 77.27%), D (n = 6, 13.64%), and B (n = 4, 9.09%).

Table 1 and Supplementary S1 present the characteristics of all included studies. A thematic synthesis was undertaken to integrate common dimensions and patterns across heterogeneous designs. The process led to the identification of five overarching themes that define the attributes of CNCP patients: (1) biological aspects; (2) functioning and quality of life; (3) psychological and mental factors; (4) social support and peer relationships; (5) social and gender determinants.

### 3.2. Biological Aspects

Multiple studies have highlighted the importance of biological processes in CNCP in addition to its intensity. Their interaction with multimorbidity and the coexistence of chronic diseases constitutes a key determinant in the clinical expression of pain. Localized CNCP was identified as a cause of increased risk of suffering cardiovascular diseases, a relatively higher risk when dealing with generalized CNCP [56]. Another study developed by Chen et al. [34] documented an increase in the prevalence of CNCP from 33% in 2011 to 58% in 2020, with an association with chronic conditions such as arthritis, arterial hypertension, diabetes, and dyslipidemia [35].

At the same time, Neba et al. [50] found that 68% of individuals with CNCP presented multimorbidity of other diseases, which hindered adherence to self-care strategies and was associated with a higher probability of opioid use [50]. In this sense, regarding medication consumption, the pharmacological management of people with CNCP reflects notable differences compared to those who do not suffer from it. This is reflected, in the first place, in figures such as those reported by Allen-Watts et al. [28], who found that 88% of the adults with CNCP studied used some type of medication, mainly non-steroidal anti-inflammatory drugs (NSAIDs) and antidepressants [28]. These findings point out that CNCP not only harms the person due to its intensity, but also compromises long-term physical health. It was also found that suffering from CNCP was associated with higher healthcare costs, particularly for medical consultations in primary health care services, finding that 50% of the people analyzed required three or more visits in a time period of three months [68].

Regarding sleep quality, sleep disorders represent a complex component in the experience of CNCP given its bidirectional relationship with it, affecting both physical functionality and emotional well-being. On one hand, several studies associate worse sleep quality with a reduction in mobility and a deterioration in the performance of activities of daily living [30,31,52,58,63]. On the other hand, from an emotional point of view, rest interrupted by pain is linked to alterations in emotional health [67], altering social and family relationships [45].

### 3.3. Functioning and Quality of Life

Findings from the scientific literature are consistent in highlighting the role of CNCP in limitations in mobility, physical functioning, and quality of life. Musculoskeletal CNCP has been associated with a significant decline in physical capacity, strength, balance, and gait [60]. In line with these findings, Rosa et al. [57] reported that certain pain typologies, such as shoulder pain, did not significantly reduce the number of daily steps but did impair mobility by 14%, emphasizing the emotional consequences of functional limitation [57].

Other pain locations, such as temporomandibular disorder (TMD), have been associated with fear of movement, or kinesiophobia, suggesting that fear of movement may restrict mobility and perpetuate the cycle of inactivity and disability characteristic of CNCP [66]. Dueñas et al. [5] identified three levels of functional limitation related to CNCP (low: 47.6%; moderate: 34.3%; and high: 18.1%), which were in turn associated with older age, longer pain duration and intensity, and poorer general health status [5].

These findings are consistent with those of other authors who have linked CNCP to poorer quality of life [30,42,56,66]. According to Mun et al. [41], CNCP exerts a profound impact on individuals, affecting not only physical well-being but also psychological and social domains, with particularly pronounced effects in the latter two [41].

### 3.4. Psychological and Mental Factors

Psychological factors play a crucial role in the experience and perception of CNCP. Among these, resilience has been identified as a key protective factor, reducing the interference of pain in daily life [27]. Damsgård et al. [36] likewise found that resilience functions as both a cultural and psychological protective factor, enhancing coping capacity and mitigating the impact of painful symptoms [31,36]. In addition, low resilience has been associated with reduced mobility and greater functional limitation [57]. In this regard, negative coping strategies such as kinesiophobia have been significantly associated with maladaptive coping mechanisms that limit mobility and perpetuate disability related to CNCP [38,66]. Pain catastrophizing has been linked to higher pain intensity, poorer mental health, and increased use of health care services [12].

Similarly, affective disorders represent another critical dimension in the relationship between mental health and CNCP. Depression plays a central role, influencing both clinical response and functional outcomes [30]. In addition, Dueñas et al. [5] reported that depressed mood was associated with greater functional disability, while the coexistence of multiple pain conditions further increased levels of depression and anxiety, highlighting the additional emotional burden of pain-related multimorbidity [41]. Likewise, individuals with TMD exhibited poorer health-related quality of life and a higher prevalence of depressive and anxiety symptoms [40,66]. Conversely, better mental health facilitated engagement in self-management activities, whereas anxiety and depression acted as factors that complicated prognosis [33].

### 3.5. Social Support and Peer Relationships

Interpersonal relationships, whether familial, marital, or friendships, play a crucial role in shaping the experience and impact of CNCP. Social and community support has been identified as a key protective factor against CNCP, with close kinship ties and extended family networks functioning as naturally embedded resilience mechanisms [36]. In this regard, higher levels of social support have been associated with a lower prevalence of persistent pain, reduced pain intensity, and greater levels of physical activity [36,63].

Marital status also appears to influence vulnerability to CNCP, with divorced or widowed individuals showing higher rates of persistent pain, whereas those who are married exhibit a lower incidence of new pain cases. This supports the hypothesis that marriage may exert a protective effect through marital bonds [53]. At the same time, CNCP can generate conflict within intimate relationships. In this context, a study conducted by Marini et al. [44] found that poor sleep quality and relationship strain were associated with greater morning irritability and increased marital conflict in couples in which one partner experienced CNCP due to osteoarthritis. This finding points to the role of relational dynamics in shaping emotional responses to CNCP [44].

### 3.6. Social and Gender Determinants

Educational attainment, employment status, and income level contribute to unequal experiences of CNCP. Regarding education, lower educational levels have been linked to a higher prevalence of CNCP [42,52], particularly in the case of TMD [40]. Conversely, higher educational attainment has been associated with a significant reduction in pain intensity [37]. Living in economically disadvantaged areas has been linked to poorer sleep quality and greater pain severity [58]. Area-level deprivation indices and residence in areas with a low Human Development Index significantly increased the likelihood of experiencing CNCP [35,52].

Occupational and work-related factors further shape CNCP outcomes. From a multidimensional perspective, Nogueira-Carrer et al. [51] found that earning an income below the minimum wage was associated with longer pain duration, poorer prognosis, and persistence of functional limitations [51]. However, this relationship remains complex. Other studies suggest that both high and low socioeconomic groups may be at risk. That is, individuals with higher education or income levels have also reported a higher prevalence of CNCP in certain contexts, possibly reflecting occupational strain or psychosocial stressors related to professional roles [42,51]. Studies by Chen et al. [34] and Vallin et al. [66] have similarly reported higher prevalence rates of musculoskeletal pain and TMD, respectively.

Individuals experiencing pain at any anatomical location showed a higher likelihood of retirement due to poor health [43]. According to Saes-Silva et al. [59], 31% of individuals with lumbar CNCP were absent from work, and 68% consulted a physician due to symptoms over a 12-month period.

Regarding geography, pain prevalence tends to be higher in rural settings [65]. Evidence indicates a progressive increase in CNCP and high-impact pain, particularly among adults aged 45–65 years living in rural and peri-urban areas [64,65]. According to Kossi et al. [42], individuals in rural populations are more likely to report lumbar CNCP.

Finally, gender differences are also reflected in the lived experience of CNCP. Gender emerges as a consistent and cross-cutting determinant. Women exhibit higher rates of musculoskeletal pain and greater functional limitation [51,52]. In addition, women tend to report poorer physical functioning and higher levels of depression, while gender-based discrimination exacerbates the interference of CNCP in daily life [32,35]. Indeed, a study by Arman et al. [29] found that women perceived pain as a form of bodily protest, which they related to years of overexertion and neglect of their own self-care.

### 3.7. Biopsychosocial Integration of Chronic Non-Cancer Pain

The integrative synthesis of the evidence included in this review consolidates chronic non-cancer pain (CNCP) as a dynamic, multidimensional, and socially embedded condition that is most appropriately understood through the biopsychosocial model [69]. Rather than resulting from isolated biological dysfunction, CNCP emerges from the continuous and reciprocal interaction among biological vulnerability, psychological regulation, functional capacity, and social context. This perspective aligns with contemporary pain science, which recognizes chronic pain as a complex adaptive process shaped by both physiological and experiential factors [9,70].

From a biological standpoint, multimorbidity, sleep disturbance, and persistent symptom burden form a fundamental substrate that contributes to pain persistence and clinical complexity. However, the evidence consistently indicates that biological mechanisms alone are insufficient to explain variability in pain trajectories or functional outcomes. Instead, biological stressors interact bidirectionally with psychological processes. Emotional distress, depressive symptomatology, and maladaptive cognitive responses, particularly catastrophizing and fear-avoidance, intensify pain perception, promote behavioral restriction, and accelerate functional decline [30,38,66]. Within this framework, the fear-avoidance model provides a robust explanatory lens, suggesting that pain-related fear leads to activity avoidance, progressive deconditioning, and disability, thereby sustaining the chronic pain cycle [71,72].

Functional limitations emerge as a central pathway through which CNCP exerts its impact on quality of life. Reduced mobility, loss of autonomy, and progressive physical deconditioning reinforce a self-perpetuating cycle of pain, inactivity, and disability [5,57]. Importantly, contemporary biopsychosocial approaches emphasize that functional outcomes are not determined solely by physical impairment but are strongly influenced by resilience, behavioral adaptation, coping flexibility, and psychological regulation [27,58].

Beyond individual-level processes, the findings highlight the structural and relational embedding of CNCP within broader social determinants of health. Socioeconomic disadvantages, limited access to resources, and environmental constraints are consistently associated with greater pain burden, poorer functional outcomes, and higher healthcare utilization [42,51,55]. At the interpersonal level, social support operates as a protective factor, mitigating emotional distress and reducing pain interference, whereas relational strain, social isolation, and reduced social capital amplify psychological burden and disability [12,36]. Gender emerges as a cross-cutting and intersectional determinant, with women consistently reporting higher pain prevalence, greater functional limitation, and stronger psychosocial impact [32,52].

Taken together, the evidence supports a biopsychosocial model of CNCP that is dynamic, interactive, and socially contextualized. Within this model, biological vulnerability, psychological processes, functional capacity, and social environment do not operate independently but continuously shape one another over time. This integrative perspective reinforces the need for multidimensional assessment and comprehensive management strategies that extend beyond symptom reduction to include psychological well-being, functional restoration, and the social conditions influencing pain trajectories. Such an approach is consistent with contemporary interdisciplinary and person-centered models of chronic pain care [9,73].

## 4. Discussion

This review integrated and analyzed the available evidence indicating that individuals living with CNCP constitute a clinically and socially complex entity, defined by the dynamic interaction of biological, psychological, social, and cultural factors. Rather than operating as separate domains, these dimensions appear to interact to produce a fluid and multi-dimensional experience of chronic pain, linking physiological processes with emotional states, coping behaviors, and social contexts [9,73].

From a biological and clinical perspective, central sensitization, low-grade chronic inflammation, and alterations in descending pain modulation systems are increasingly recognized as key pathophysiological processes in CNCP [74,75]. However, contemporary scientific evidence highlights that although biological mechanisms are fundamental to understanding pain onset, they are insufficient to explain its chronification or the degree of disability it produces [76]. This is consistent with previous reviews suggesting that biological correlations explain less than half of the variance in functional outcomes, whereas psychosocial variables, such as resilience, depression, and social support, demonstrate stronger predictive power [77].

In this context, healthcare professionals play a key role in comprehensive assessment and in identifying biological and behavioral risk factors. Symptom monitoring, education on the rational use of analgesics, and the promotion of healthy lifestyles, including regular physical activity, balanced nutrition, and restorative sleep, constitute cost-effective interventions that help prevent exacerbations and improve clinical outcomes [78]. Moreover, these strategies enhance patient autonomy and support more sustainable long-term pain management [79].

Psychological factors are among the most consistent determinants of the course and expression of CP. Numerous studies have shown that depression, anxiety, and catastrophizing act as pain amplifiers and predictors of disability [80]. These emotional disturbances not only alter sensory perception but also interfere with motivation, treatment adherence, and social participation. Conversely, resilience has been established as a protective factor associated with better functional adaptation, lower pain interference in daily life, and higher quality of life [81]. This evidence converges with previous meta-analyses highlighting that cognitive and emotional regulation mechanisms mediate the relationship between pain intensity and functionality, suggesting that psychological adaptation is a key determinant of recovery rather than a secondary consequence [77].

Recent evidence supports the effectiveness of cognitive–behavioral interventions and acceptance and commitment therapy, both aimed at promoting self-management and emotional regulation. The incorporation of psychoeducational strategies into clinical management, such as early identification of depressive symptoms and the teaching of relaxation and active coping techniques, may reduce emotional burden and improve functional outcomes [82]. From the disciplinary perspective of nursing, the prominence of psychological distress, coping strategies, and functional limitation identified across the reviewed studies supports recognizing care as a relational and educational process that facilitates adaptation and self-efficacy. Person-centered nursing frameworks, such as the Self-Care and Adaptation models, provide conceptual tools for integrating emotional, behavioral, and environmental dimensions into pain management [83,84].

The social dimension of CNCP has also gained increasing relevance, particularly within the framework of the social determinants of health. Socioeconomic disadvantage and job insecurity have been associated with greater incidence and severity of pain [5,85]. In addition, lack of social support and perceived loneliness amplify the subjective experience of suffering and increase the risk of functional decline [86]. These findings underscore the need to include assessment of the social environment, support networks, and barriers to healthcare access as part of routine clinical evaluations. Comparatively, our synthesis aligns with previous studies identifying social deprivation and isolation as among the strongest predictors of high-impact CNCP, independent of biological status [87].

From a public health perspective, addressing CP requires a broader response that extends beyond the individual clinical setting. Strengthening intersectoral policies aimed at reducing poverty, improving access to healthcare, and promoting healthy community environments may significantly contribute to the prevention and management of persistent pain [88].

This integrative review presents several limitations that should be considered when interpreting the findings. First, most included studies were cross-sectional or observational in design, which restricts causal inference and limits understanding of temporal dynamics in the evolution of chronic non-cancer pain. Furthermore, the limited number of experimental and longitudinal studies reduces the ability to accurately assess the direction and magnitude of observed associations [17,89].

Second, methodological heterogeneity across studies, in terms of design, populations, operational definitions of chronic pain, measurement instruments, and analyzed variables, complicated direct comparison and uniform synthesis of results. This variability is inherent to integrative reviews, which incorporate multiple forms of evidence, but may influence the consistency and generalizability of conclusions [17,24].

Another relevant limitation is the predominance of studies conducted in high-income countries, which may restrict the transferability of findings to different sociocultural contexts, particularly in low- and middle-income settings where social and structural determinants of pain may manifest differently [11,73]. Similarly, the limited inclusion of qualitative studies may have reduced the interpretative depth regarding the subjective experience of pain and its sociocultural dimensions, an aspect identified as relevant in integrative reviews addressing complex health phenomena [17]. Additionally, only studies published in English and Spanish were included, which may have introduced language bias and potentially excluded relevant evidence published in other languages [90].

The findings of this review highlight the need to critically re-examine the prevailing care model. Although academic discourse increasingly recognizes the multidimensional nature of CNCP, clinical practice remains largely biomedical and fragmented, emphasizing pharmacological management with limited interdisciplinary coordination. Available evidence supports the effectiveness of integrated care models that combine multidimensional assessment, health education, and continuity of care. Such models, implemented across diverse healthcare systems, appear to have demonstrated improvements in functional outcomes, reductions in emotional distress, and decreased reliance on opioid therapy [91,92,93].

At the population level, CNCP should be recognized as a major public health concern due to its high prevalence, associated disability burden, and substantial social and economic costs [4]. Integrating CP management into primary care and community health programs, grounded in principles of equity and gender sensitivity, represents a fundamental strategy to mitigate its population-level impact [94].

## 5. Conclusions

This integrative review synthesizes current evidence showing that chronic non-cancer pain is a multidimensional condition shaped by interacting biological, psychological, and social determinants that influence functioning, quality of life, and healthcare utilization. Biological mechanisms contribute to pain persistence, and psychological distress, coping patterns, and social conditions appear to play a central role in shaping functional outcomes. The findings highlight the need for multidimensional and person-centered approaches to CNCP management that integrate clinical assessment with psychological support and consideration of social context. Future research should prioritize longitudinal and intervention studies to better clarify causal pathways and inform integrated care strategies.

## Figures and Tables

**Table 1:** Summary of Findings.

Author, Year, Region	Design, Intervention	Aim	Concept	Summary of Findings	Measurements
Åkerblom et al., 2025 [27] Sweden	Observational cohort (long-term follow-up) Level 2+ Grade C	To examine long-term clinical and health economic outcomes of multidisciplinary CBT for CP, and to explore whether psychological inflexibility and patient characteristics predicted or mediated these outcomes.	CP defined as pain persisting or recurring for ≥3 months.	Improvements in pain, depression, and interference were sustained for 3 years; psychological inflexibility was identified as the primary mediator and a predictor of long-term depressive symptoms and sick leave.	NRS, 0–10 MPI-2 HADS-D PIPS Swedish national registries
Allen-Watts et al., 2022 [28] UK	Cross-sectional survey Level 2+ Grade C	To investigate prevalence and predictors of CWP	CWP defined as pain lasting ≥3 months in multiple body regions	CWP prevalence was 12.7%, significantly impacting daily functioning. Key predictors included female sex, advanced age, low physical activity, and psychological distress.	Structured self-reported questionnaires HADS
Arman et al., 2020 [29] Sweden	Qualitative study, Gadamerian philosophical hermeneutics Level 4 Grade D	To understand the lived experience of women with CP from a caring science and gender perspective	CP in women, interpreted through lived experiences considering gender relations and cultural context	Women described an overwhelming life situation characterized by overexertion and loneliness. Suffering is deeply intertwined with gender roles, cultural expectations, and mental health comorbidities.	In-depth qualitative interviews
Barron et al., 2024 [30] UK	Cross-sectional, population-based analysis Level 2+ Grade C	To define biologically grounded, function-based pain profiles and assess their clinical and healthcare delivery relevance compared with the conventional body-part framework.	Pain experience defined from 154 pain-related variables, integrated with 100 brain volume measures.	CP was identified as a multidimensional phenomenon manifesting in four unique profiles. These are more closely linked to modifiable chronic diseases and lifestyle factors than to specific anatomical locations.	Pain experience questionnaire items from UK Biobank BPI PHQ-9
Bartley et al., 2022 [31] USA	Cross-sectional, resilience framework Level 2− Grade D	To examine whether phenotypic profiles of resilience among older adults with CLBP differ across sleep disturbance, fatigue, and cognitive abilities	CLBP defined as pain lasting ≥3 months, with moderate intensity on at least half of the days during the preceding 3 months.	High resilience correlated with superior cognitive function and lower fatigue. Conversely, low income, obesity, and minority racial status were associated with lower psychosocial resilience and poorer clinical outcomes.	NRS, 0–10. PANAS ADHS PROMIS Positive Affect and Well-Being Scale LOT-R PROMIS Support scales, Anthropometric variables
Boring et al., 2025 [32] USA	Cross-sectional, secondary analysis of the MIDUS Level 2+ Grade C	To examine whether daily discrimination in general, and gender discrimination specifically, are associated with greater pain interference, and whether these effects differ by sex.	CP assessed by self-report: pain persisting beyond normal healing time, lasting from a few months to many years.	Discrimination functions as a chronic stressor that significantly increases pain interference exclusively in women, highlighting social determinants of sex disparities in health.	Pain interference 0–10 scale. 9 items, MIDUS. BMI, clinical variables
Budge et al., 2020 [33] New Zealand	Secondary analysis of the Talking about Health study Level 3 Grade D	To examine how well people with long-term conditions make use of self-management strategies to control their pain	CP is a common, yet often overlooked, long-term condition.	CP frequently co-occurs with anxiety and sleep disorders. Notably, one in five affected individuals does not seek medical help, emphasizing the critical role of self-management in primary care.	PROMIS PAM A custom questionnaire with questions on general health, long-term conditions, pain
Chen et al., 2025 [34] China	Cross-sectional study Level 2+ Grade C	To estimate prevalence and psychosocial correlates of CLBP	CLBP defined as pain persisting for ≥3 months in the lower back	A 21.9% prevalence of CLBP was found, which is strongly correlated with significant functional disability, depression, anxiety, and low social support.	HADS
Cheng et al., 2022 [35] China	Cross-sectional survey Level 2+ Grade C	To investigate prevalence and sociodemographic correlates of CP	CP defined as pain lasting ≥3 months	Prevalence reached 30.1%, with a high impact on QoL exacerbated by advanced age, female sex, and lower educational attainment.	WHOQOL-BREF
Damsgård et al., 2020 [36] Norway	Prospective cohort study Level 2+ Grade C	To evaluate predictors of return to work in patients with MSP	MSP defined as pain lasting ≥3 months	59% of patients achieved RTW within one year. Success was predicted by high self-efficacy, lower initial pain intensity, and fewer comorbidities.	BPI; self-efficacy and psychosocial scales; work outcomes monitored longitudinally
Dueñas et al., 2019 [5] Spain	Nationwide cross-sectional epidemiological study Level 2+ Grade C	To establish subgroups of people with CP based on limitations in ADLs, and to identify sociodemographic, pain-related, and psychosocial variables associated with each subgroup	CP: pain present at least 4–5 days per week during the preceding 3 months.	Greater pain intensity and duration were linked to severe functional limitations, leading to higher rates of job loss, social isolation, and elevated anxiety/depression.	Difficulty in walking, lifting, bathing, dressing, housework, sleeping, etc. Intensity, duration, location, medication use. Sadness, anxiety, Sociodemographics
Eilayyan et al., 2025 [37] Canada	Secondary analysis of a longitudinal study Level 2+ Grade C	To estimate the relationships between pain intensity, psychological distress, self-efficacy, functional ability, and healthcare utilization among individuals with CLBP	There is evidence for CP to be classified as a neurological disease	QoL and healthcare utilization are more heavily influenced by the patient’s beliefs about pain and self-efficacy than by the physical intensity of the symptoms.	BPI SF-12 PHQ-9 HADS Self-Efficacy Scale ODI
Ferreira Valente et al., 2024 [38] Portugal	Cross-sectional study Level 2+ Grade C	To examine associations between mental pain and CP severity	CP defined as pain persisting for ≥3 months.	“Mental pain” emerged as an independent factor explaining significant variance in CP severity, beyond the contributions of traditional depression or anxiety.	BPI OMMP TMPS HADS WHOQOL-BREF
Fong et al., 2024 [39] China	Cross-sectional, non-randomized, observational study Level 3 Grade D	To identify the significant physical, psychological, and social determinants associated with EuroQol-5D among Chinese older people with MSP	MSP is a very common condition that has a significant impact on people worldwide	MSP severely impairs physical and social domains in older adults, with QoL being determined by the number of painful regions, marital status, and social welfare.	BMI BPI PHQ-9 GAD-7 EQ-5D
Heikkinen et al., 2024 [40] Finland	Cross-sectional analysis Level 2+ Grade C	To evaluate prevalence of TMD and associations with sociodemographic and psychosocial factors	TMD diagnosed using the Diagnostic Criteria for TMD.	30.4% reported symptoms of TMD, which were consistently associated with female sex and anxiety/depression symptoms.	HSCL-25 GAD-7
Mun et al., 2019 [41] USA	Cross-sectional, observational study Level 2+ Grade C	To examine how multiple CP conditions and multiple pain sites are associated with sociodemographics.	CP defined as consistent pain severity, interference, and/or emotional burden for ≥6 months.	88.6% of patients reported multiple pain sites, associated with higher catastrophizing and central sensitization stemming from low social resources.	PCP:S PCP:EA CES-D
Karran et al., 2022 [12] Australia	Mixed-methods convergent parallel design Level 2+ Grade C	To explore the role of social determinants of health in health outcomes, healthcare experiences, and perceived barriers/facilitators to care in socioeconomically disadvantaged adults with CP.	CP defined as pain occurring daily for ≥3 months.	Social isolation and financial instability strongly correlate with higher pain interference, worsened by a clinical focus that is often excessively biomedical and ignores social needs.	WHYMPI K-10 WHOQOL-BREF. FCI CAGE-AID. SHS Tool. IRSAD MMM
Kossi et al., 2022 [42] Finland	Cross-sectional population-based study Level 2+ Grade C	To investigate prevalence and risk factors of CP	CP defined as pain lasting ≥3 months	A 30.8% prevalence was reported, linked to low socioeconomic status and significantly higher healthcare utilization.	Structured interviews; sociodemographic and health questionnaires
Lee et al., 2020 [43] South Korea	Longitudinal analysis Level 2+ Grade C	To examine relationship between MSP and IHR and the modifying role of socioeconomic status	MSP at multiple sites categorized by severity.	Multiple-site MSP significantly increases productivity losses, with a more pronounced effect in older white-collar workers and high-income groups.	Age, BMI, smoking, history of disease, job category, and annual income
Marini et al., 2020 [44] Italy	Cross-sectional survey Level 2+ Grade C	To investigate prevalence of CP and associated psychosocial factors	CP defined as pain persisting for ≥3 months, interfering with daily life	28% of adults suffer from CP, with the functional and mental burden being significantly higher for those with low income and reduced educational levels.	Self-reported questionnaires on CP duration, sociodemographic data, psychosocial status
McQueenie et al., 2021 [45] UK	Longitudinal cohort study Level 2+ Grade C	To evaluate effects of multimorbidity on CP outcomes	CP defined as pain lasting ≥3 months in any site	The presence of multiple chronic conditions acts as an aggravating factor that worsens disability and drastically increases medical resource consumption.	CP and multimorbidity assessed via self-reports and medical records GCPS
Moreno-Ligero et al., 2024 [46] Spain	Cross-sectional study Level 2− Grade D	To identify factors associated with pain-related functional interference in people with CLBP	CLBP defined as pain persisting for ≥3 months.	35.4% of patients reported high interference, which was linked to obesity, poor sleep quality, and the use of weak opioids.	NRS PPIQ TUG IPAQ-SF. MOS. HADS. Duke-UNC HRQoL SF-12
Najafi et al., 2023 [47] Iran	Cross-sectional study Level 2+ Grade C	To assess prevalence and correlates of CP among older adults	CP defined as pain lasting ≥3 months in any body site	Prevalence reached 53% in the elderly, associated with significant functional impairment and comorbidities such as diabetes and hypertension.	Structured questionnaires, physical health assessments
Nahin et al., 2021 [48] USA	Cross-sectional, nationally representative study Level 2+ Grade C	To provide national surveillance estimates of pain prevalence, chronicity, severity, and impact considering race/ethnicity interactions	CP pain on most/every day in past 6 months. High-impact CP: CP limiting life or work activities most/every day in past 6 months.	Significant ethnic disparities were identified; Puerto Ricans exhibited the highest prevalence of high-impact chronic pain compared to other Hispanic groups.	Washington Group pain questions (2010–2015). CP, high-impact CP, Category 3–4 pain. Sociodemographics
Nduwimana et al., 2022 [49] Burundi	Cross-sectional, non-randomized, observational study Level 3 Grade D	To investigate the biopsychosocial factors that influence the CLBP-related activity limitations.	CLBP is an increasing burden worldwide.	Limitations in activities are conditioned by educational level, healthcare coverage, physical fitness, and the presence of depression.	BDI-II FABQ RMDQ
Neba et al., 2024 [50] US	Cross-sectional, nationally representative study Level 2+ Grade C	To estimate prevalence and types of pain management strategies among adults with multimorbidity and CP, and analyse associations with sociodemographic, lifestyle, and clinical factors.	CP: pain on some/most/every day in the past 3 months. Multimorbidity: ≥2 chronic conditions	68% of adults with CP have multimorbidity. Food insecurity and low income act as barriers to multimodal care, often leading to a higher reliance on opioid therapy.	Pain severity treatment type Sociodemographics
Nogueira Carrer et al., 2024 [51] Brazil	Cross-sectional study Level 2+ Grade C	To examine prevalence and correlates of CP	CP defined as pain persisting for ≥3 months	A 25.7% prevalence was recorded, where unemployment and comorbid chronic diseases were the primary factors diminishing quality of life.	WHOQOL-BREF
Oliveira et al., 2023 [52] Portugal	Cross-sectional study Level 2+ Grade C	To assess prevalence of CLBP	CLBP defined as pain persisting for ≥12 weeks in the lower back	Prevalence of CLBP was 19.3%, with substantial disability determined by obesity, a sedentary lifestyle, and low education.	IPAQ
Peace et al., 2023 [53] USA	Cross-sectional survey Level 2+ Grade C	To examine associations between CP severity and quality of life	CP defined as pain persisting for ≥3 months in any body site	This research established critical associations between CP severity and quality of life; findings indicated that greater CP severity is directly linked to poorer quality of life and a significant reduction in daily functioning	BPI WHOQOL-BREF
Peat et al., 2020 [54] UK	Longitudinal cohort study Level 2++ Grade B	To investigate prognosis of chronic knee pain and its impact on disability	Chronic knee pain defined as pain on most days for ≥3 months	The investigation focused on the prognosis and disability associated with CP in knee; results demonstrated that female sex, obesity, depression, and low physical activity are key factors leading to persistent disability and increased healthcare utilization	Self-reported questionnaires (pain intensity, WOMAC; clinical assessments
Rassu et al., 2025 [55] USA	Cross-sectional study Level 2+ Grade C	To investigate whether neighborhood disadvantage is associated with pain intensity, fatigue, emotional distress, and ADI, and whether catastrophizing and fear mediate these relationships.	CP defined by clinical presentation in patients referred for psychological evaluation of CP conditions	The study identified that a higher ADI correlates with increased pain intensity, fatigue, and emotional distress; findings highlighted neighborhood disadvantage as a social determinant of worse pain outcomes, with pain catastrophizing and fear serving as the primary mediators between social context and pain burden	PCOQ. PCS. TSK-13. Age, gender.
Rönnegård et al., 2022 [56] Sweden	Cross-sectional population-based study Level 2+ Grade C	To assess prevalence and determinants of CP	CP defined as pain lasting ≥3 months	This study assessed the prevalence and determinants of CP; it reported a 29.4% prevalence rate and established that female sex, older age, low socioeconomic status, and comorbidities are significantly associated with higher prevalence, disability, and healthcare use	Structured interviews and health questionnaires; sociodemographic data
Rosa et al., 2025 [57] Brazil	Cross-sectional study Level 2+ Grade C	To estimate prevalence of CMP and associated sociodemographic factors	CMP defined as pain in muscles or joints lasting ≥3 months	The analysis explored the prevalence of CMP and its sociodemographic determinants; findings revealed a 27.3% prevalence rate, with female sex, advanced age, lower education, and low income contributing to a substantial disability burden	Structured questionnaires, sociodemographic survey, logistic regression analysis
Rumble et al., 2021 [58] USA	Longitudinal cohort study Level 2++ Grade B	To examine the relationship between resilience and pain outcomes in CLBP	CLBP defined as pain persisting for ≥3 months in the lower back	This research evaluated the role of resilience in CLBP outcomes; findings suggested that higher resilience acts as a protective factor against adverse outcomes, correlating with lower pain intensity, reduced disability, and improved quality of life	Patient-reported outcomes, validated resilience scales, pain questionnaires
Saes-Silva et al., 2021 [59] Brazil	Population-based cross-sectional study Level 2+ Grade C	To determine the prevalence of CBP, associated factors, health service use, work absenteeism, and impact on health outcomes.	CBP defined as pain lasting ≥3 consecutive months in cervical, thoracic, or lumbar regions during the last year.	The study established a CBP prevalence of 20.7%, associated with female sex, smoking, obesity, and high stress; results significantly linked CBP to poor sleep quality, worse self-rated health, and depressive symptoms, noting that eliminating CBP could reduce poor health perceptions by 25%	Structured questionnaire Health outcomes: sleep quality, self-rated health, WHOQOL, PHQ-9, faces scale. Sociodemographics
Sardina et al., 2022 [60] Italy	Cross-sectional study Level 2+ Grade C	To investigate prevalence and correlates of CP in adolescents	CP defined as pain lasting ≥3 months in any site	Investigating the prevalence and correlates of CP in adolescents, this study reported an 18.7% prevalence rate; it identified female sex, psychological distress, and family conflict as factors that adversely impact school attendance and social functioning	Self-reported questionnaires on pain duration, psychosocial measures, school functioning
Saba et al., 2024 [61] USA	Cross-sectional survey Level 2+ Grade C	To evaluate CP prevalence and associated health behaviors	CP defined as pain persisting for ≥3 months in any body region	This evaluation of CP prevalence and health behaviors identified a 20.1% prevalence rate; the findings highlighted strong associations between CP and modifiable risk behaviors, including smoking, alcohol consumption, and low physical activity	Structured health survey
Strath et al., 2024 [62] USA	Cross-sectional analysis of National Health Interview Survey Level 2++ Grade B	To examine prevalence of CP and high-impact CP in US adults	CP defined as pain most days or every day during past 3 months; high-impact CP defined as CP limiting daily activities	This national analysis identified a significant burden for both CP (20.4%) and high-impact CP (7.4%); findings showed that female sex, older age, low income, and comorbid conditions are associated with higher prevalence and impact	National Health Interview Survey; descriptive statistics, regression analyses
Stevens et al., 2020 [63] Canada	Cross-sectional survey Level 2+ Grade C	To examine prevalence and correlates of MSP	MSP defined as pain lasting ≥3 months in muscles or joints	The research assessed the prevalence and correlates of MSP, reporting a 33.2% prevalence rate; results identified significant impacts on work productivity and quality of life associated with female sex, low income, and depression	HADS
Sun et al., 2025 [64] USA	Secondary analysis of the National Health Interview Survey Longitudinal Cohort Level 2+ Grade C	To examine pain prevalences and longitudinal transitions across the rural–urban continuum	CP is pain lasting more than 3 months.	Findings demonstrated that CP and high-impact CP prevalence systematically increase along the rural–urban continuum; rural residency and socioeconomic disadvantage were linked to a higher likelihood of pain progression, increased disability, and a lower probability of recovery	National Health Interview Survey Longitudinal Sociodemographics Pain incidence
Tinoco et al., 2024 [65] Brazil	Cross-sectional population-based survey Level 2++ Grade B	To estimate prevalence and correlates of CP in Brazil	CP defined as pain lasting ≥3 months, self-reported	This population-based survey estimated a 29.5% CP prevalence in Brazil; it established that female sex, lower education, unemployment, and chronic diseases are associated with CP and its negative impact on work capacity and quality of life	Household-based interviews, standardized questionnaires, regression analyses
Vallin et al., 2024 [66] France	Cross-sectional study Level 2+ Grade C	To examine association between CLBP and mental health outcomes	CLBP defined as pain persisting for ≥3 months in the lower back	The study examined the associations between CLBP and mental health, demonstrating that depression and anxiety are linked to greater disability; findings indicated that CLBP significantly impairs quality of life and increases the risk of depressive symptoms	BPI HADS
Yu et al., 2020 [67] China	Cross-sectional study Level 2+ Grade C	To investigate prevalence of CP and its impact on mental health	CP defined as pain lasting ≥3 months in any body part	This investigation into the prevalence and mental health correlates of CP reported a 46% prevalence rate; results established that female sex, depression, comorbidities, and low income are significantly linked to a higher risk of depressive symptoms	Self-administered questionnaires GDS
Zanuto et al., 2021 [68] Brazil	Cross-sectional population-based study Level 2+ Grade C	To estimate prevalence of CP and associated sociodemographic factors	CP defined as pain lasting ≥3 months, self-reported	This study estimated a 27% CP prevalence and its sociodemographic correlates; findings showed that female sex, older age, low education, and comorbidities place a significant burden on daily living and healthcare utilization	Household interviews; structured questionnaires

Note. Authors’ own elaboration. References sorted by authors’ surnames in English alphabetical order (A to Z). ADHS: Adult Dispositional Hope Scale; ADI: Area Deprivation Index; ADLs: Activities of Daily Living; BDI-II: Beck Depression Inventory-II; BMI: Body Mass Index; BPI: Brief Pain Inventory; CBP: Chronic Back Pain; CES-D: Center for Epidemiologic Studies Depression Scale; CLBP: Chronic Low Back Pain; EQ-5D: EuroQol-5 Dimension Questionnaire; FCI: Functional Comorbidity Index; FABQ: Fear-Avoidance Beliefs Questionnaire; GCPS: Graded Chronic Pain Scale; GDS: Geriatric Depression Scale; GAD-7: Generalized Anxiety Disorder-7; HADS: Hospital Anxiety and Depression Scale; HRQoL: Health-Related Quality of Life; HSCL-25: Hopkins Symptom Checklist-25; IPAQ: International Physical Activity Questionnaire; IPAQ-SF: International Physical Activity Questionnaire-Short Form; IHR: Ill-Health Retirement; IRSAD: Index of Relative Socio-Economic Advantage and Disadvantage; K-10: Kessler Psychological Distress Scale; LOT-R: Life Orientation Test–Revised; MIDUS: Midlife in the United States study; MMM: Modified Monash Model; MOS: Medical Outcomes Study; MPI-2: Multidimensional Pain Inventory; MSP: Musculoskeletal Pain; NRS: Numerical Rating Scale; OMMP: Orbach and Mikulincer Mental Pain Scale; ODI: Oswestry Disability Index; PAM: Patient Activation Measure; PCS: Pain Catastrophizing Scale; PCOQ: Patient-Centered Outcomes Questionnaire; PCP:S/PCP:EA: Profile of Chronic Pain-Screen/Extended Assessment; PHQ-9: Patient Health Questionnaire-9; PPIQ: Pictorial Pain Interference Questionnaire; PROMIS: Patient-Reported Outcomes Measurement Information System; QoL: Quality of Life; RMDQ: Roland Morris Disability Questionnaire; SF-12: 12-Item Short Form Health Survey; SHS: Social History Screening Tool; TMD: Temporomandibular Disorders; TMPS: Tolerance for Mental Pain Scale; TSK/TSK-13: Tampa Scale of Kinesiophobia; TUG: Timed Up and Go Test; UK Biobank: United Kingdom Biobank study; WHOQOL-BREF: World Health Organization Quality of Life Questionnaire-Short Form; WHYMPI: West Haven-Yale Multidimensional Pain Inventory; WOMAC: Western Ontario and McMaster Universities Osteoarthritis Index.

## Data Availability

No new data were created or analyzed in this study.

## Supplementary Materials

The following supporting information can be downloaded at: https://www.mdpi.com/article/10.3390/healthcare14060725/s1.
