# Supplementary material for: Biopsychosocial and Cultural Determinants of Functioning and Healthcare Outcomes in Chronic Non-Cancer Pain: An Integrative Review
Source: Healthcare (Basel). 2026 Mar 12;14(6):725. doi: 10.3390/healthcare14060725 (PMC13026475; doi:10.3390/healthcare14060725)
Supplement: Supplementary file 1 [file healthcare-14-00725-s001.zip › healthcare-4175911-supplementary.pdf]

Supplementary 1

Key Characteristics of Included Studies

Author and year	Findings
Åkerblom et al., 2025	Improvements in pain, depression, and interference were sustained for 3 years; psychological inflexibility predicted long-term depressive symptoms and sick leave.
Allen-Watts et al., 2022	CWP prevalence was 12.7%; higher risk in women, older age, low physical activity, and psychological distress.
Arman et al., 2020	Women described chronic pain as an overwhelming life situation linked to overexertion, loneliness, gender roles, and cultural expectations.
Barron et al., 2024	Chronic pain presents four multidimensional profiles more related to chronic diseases and lifestyle factors than to anatomical location.
Bartley et al., 2022	High resilience was associated with better cognitive function and less fatigue; low income, obesity, and minority status were linked to poorer outcomes.
Boring et al., 2025	Discrimination increases pain interference in women, highlighting social determinants of sex disparities in health.
Budge et al., 2020	Chronic pain frequently co-occurs with anxiety and sleep disorders; one in five individuals does not seek medical help.
Chen et al., 2025	CLBP prevalence was 21.9%; strongly associated with disability, depression, anxiety, and low social support.
Cheng et al., 2022	CP prevalence reached 30.1%, with strong impact on quality of life, especially among older adults, women, and individuals with lower education.
Damsgård et al., 2020	59% returned to work within one year; success predicted by higher self-efficacy, lower pain intensity, and fewer comorbidities.
Dueñas et al., 2019	Greater pain intensity and duration were associated with severe functional limitations, job loss, social isolation, and anxiety/depression.
Eilayyan et al., 2025	Quality of life and healthcare utilization depend more on pain beliefs and self-efficacy than on physical pain intensity.
Ferreira Valente et al., 2024	"Mental pain" independently explains variation in chronic pain severity beyond depression or anxiety.
Fong et al., 2024	Musculoskeletal pain significantly affects physical and social functioning in older adults; QoL depends on number of painful regions and social support.
Heikkinen et al., 2024	30.4% reported TMD symptoms; strongly associated with female sex and anxiety/depression symptoms.
Mun et al., 2019	88.6% reported multiple pain sites; linked to catastrophizing and central sensitization due to low social resources.
Karran et al., 2022	Social isolation and financial instability increase pain interference; healthcare often overlooks social needs.
Kossi et al., 2022	CP prevalence was 30.8%, associated with low socioeconomic status and higher healthcare utilization.
Lee et al., 2020	Multiple-site musculoskeletal pain increases productivity loss, especially among older white-collar workers.
Marini et al., 2020	CP prevalence was 28%; higher functional and mental burden among individuals with low income and education.
McQueenie et al., 2021	Multimorbidity aggravates disability and significantly increases healthcare resource use.
Moreno-Ligero et al., 2024	35.4% reported high interference from CLBP; associated with obesity, poor sleep quality, and weak opioid use.
Najafi et al., 2023	CP prevalence reached 53% in older adults, associated with functional impairment and comorbidities such as diabetes and hypertension.
Nahin et al., 2021	Significant ethnic disparities in high-impact chronic pain; Puerto Ricans showed the highest prevalence.
Nduwimana et al., 2022	Activity limitations were associated with education level, healthcare coverage, physical fitness, and depression.
Neba et al., 2024	68% of adults with CP had multimorbidity; food insecurity and low income hinder multimodal care and increase opioid reliance.
Nogueira Carrer et al., 2024	CP prevalence was 25.7%; unemployment and chronic diseases strongly reduced quality of life.
Oliveira et al., 2023	CLBP prevalence was 19.3%; disability linked to obesity, sedentary lifestyle, and low education.
Peace et al., 2023	Greater CP severity is directly associated with poorer quality of life and reduced daily functioning.
Peat et al., 2020	Persistent disability in chronic knee pain is associated with female sex, obesity, depression, and low physical activity.
Rassu et al., 2025	Neighborhood disadvantage correlates with higher pain intensity, fatigue, and emotional distress, mediated by catastrophizing and fear.
Rönnegård et al., 2022	CP prevalence was 29.4%; higher among women, older individuals, and those with low socioeconomic status or comorbidities.

Rosa et al., 2025	CMP prevalence was 27.3%; higher disability among women, older adults, and individuals with lower education and income.
Rumble et al., 2021	Higher resilience acts as a protective factor, reducing pain intensity, disability, and improving quality of life.
Saes-Silva et al., 2021	CBP prevalence was 20.7%; associated with smoking, obesity, stress, poor sleep quality, and depressive symptoms.
Sardina et al., 2022	CP prevalence in adolescents was 18.7%; associated with female sex, psychological distress, and family conflict.
Saba et al., 2024	CP prevalence was 20.1%; strongly associated with modifiable behaviors such as smoking, alcohol consumption, and low physical activity.
Strath et al., 2024	CP prevalence was 20.4% and high-impact CP 7.4%; higher among women, older adults, low-income groups, and those with comorbidities.
Stevens et al., 2020	MSP prevalence was 33.2%, significantly affecting work productivity and quality of life.
Sun et al., 2025	CP prevalence and progression increase in rural areas and socioeconomically disadvantaged contexts.
Tinoco et al., 2024	CP prevalence was 29.5%; associated with female sex, low education, unemployment, and chronic diseases.
Vallin et al., 2024	CLBP is strongly linked to depression and anxiety, increasing disability and reducing quality of life.
Yu et al., 2020	CP prevalence was 46%; female sex, depression, comorbidities, and low income increase risk of depressive symptoms.
Zanuto et al., 2021	CP prevalence was 27%; associated with female sex, older age, low education, and comorbidities.

Note. Authors' own elaboration. CP: Chronic Pain; CWP: Chronic Widespread Pain; CLBP: Chronic Low Back Pain; MSP: Musculoskeletal Pain; CMP: Chronic Musculoskeletal Pain; CBP: Chronic Back Pain; TMD: Temporomandibular Disorders; QoL: Quality of Life; RTW: Return to Work; ADI: Area Deprivation Index
